# Comparative Effects of Vasectomy Surgery and Buprenorphine Treatment on Faecal Corticosterone Concentrations and Behaviour Assessed by Manual and Automated Analysis Methods in C57 and C3H Mice

**DOI:** 10.1371/journal.pone.0075948

**Published:** 2013-09-30

**Authors:** Sian Wright-Williams, Paul A. Flecknell, Johnny V. Roughan

**Affiliations:** Institute of Neuroscience, Newcastle University, Newcastle upon Tyne, Tyne and Wear, United Kingdom; University of Arizona, United States of America

## Abstract

Establishing effective cage-side pain assessment methods is essential if post-surgical pain is to be controlled effectively in laboratory animals. Changes to overall activity levels are the most common methods of assessment, but may not be the most appropriate for establishing the analgesic properties of drugs, especially in mice, due their high activity levels. Use of drugs that can affect activity (e.g. opioids) is also a problem. The relative merits of both manual and automated behaviour data collection methods was determined in two inbred mouse strains undergoing vasectomy following treatment with one of 2 buprenorphine dose rates. Body weights and the effects of surgery and buprenorphine on faecal corticosterone were also measured.

Surgery caused abnormal behaviour and reduced activity levels, but high dose buprenorphine caused such large-scale increases in activity in controls that we could not establish analgesic effects in surgery groups. Only pain-specific behaviour scoring using the manual approach was effective in showing 0.05 mg/kg buprenorphine alleviated post-vasectomy pain. The C57 mice also responded better to buprenorphine than C3H mice, indicating they were either less painful, or more responsive to its analgesic effects. C3H mice were more susceptible to the confounding effects of buprenorphine irrespective of whether data were collected manually or via the automated approach. Faecal corticosterone levels, although variable, were higher in untreated surgery mice than in control groups, also indicating the presence of pain or distress.

Pain-specific scoring was superior to activity monitoring for assessing the analgesic properties of buprenorphine in vasectomised mice. Buprenorphine (0.01 mg/kg), in these strains of male mice, for this procedure, provided inadequate analgesia and although 0.05 mg/kg was more effective, not completely so. The findings support the recommendation that analgesic dose rates should be adjusted in relation to the potential severity of the surgical procedure, the mouse strain, and the individual animals' response.

## Introduction

Providing an effective dose of an analgesic to prevent pain in laboratory mice first requires the development of appropriate methods to identify its presence and evaluate its intensity. Efforts to establish such methods have described use of generalised behaviour changes [1]–[3], clinical assessments [4], measurements of physiological changes such as faecal or plasma corticosterone levels [2], [3], [5], [6], or assessed heart rate and body temperature [7]. However, these have either not fully validated the parameters used to assess pain, or require specialist recording equipment or invasive methodologies (e.g. implanted telemetry devices, blood sampling). The limited success so far achieved in studies using behaviour is also due to the excessive time needed for assessments, especially if large numbers of animals are involved. Due to their highly active nature we have found this approach to be especially challenging in mice, and this is probably why more comprehensive dose recommendations for the large number of different rodent surgical models are currently not available. Efforts to address this have used automated behaviour analyses to increase the speed of data collection [1], [8]–[10]. However, this comes at the cost of needing some specialised equipment and loss of specificity regarding the individual behaviours that are included in the ethogram. Others have used methods such as nest-building and burrowing behaviour [11], both of which can be compromised by pain, or more novel parameters such as changes in facial expressions [12]–[14]. However, it remains to be established if any of these methods are superior to activity scoring in providing a means of identifying pain and the effects of analgesics in mice.

Another option is to use a ‘pain-specific’ approach whereby subtle abnormal events are scored rather than generalised activity, and we have previously demonstrated this to be highly effective in rats [15]–[17]. This simplifies the scoring process and requires minimal training [18] and so provides a practicable ‘cage-side’ method of assessing pain in either singly or group housed animals. Although we have used the pain-specific approach in vasectomised mice given different doses of meloxicam [2] or paracetamol [10], or these drugs combined [9], it was not more effective than activity monitoring. It was possible to differentiate between normal and surgery groups using pain-specific scoring, but not between groups given different dose rates of analgesics. This is a critical step in validating any proposed method of pain assessment intended for use in mice, or in fact any other species. The lack of positive effects of drug treatment reported by our group across a relatively wide range of doses of meloxicam (between 5 and 20 mg/kg [2]) has been replicated with other NSAIDs [1], [14]. This leaves the possibility that mice either require considerably higher dose rates of NSAIDs, or that they may be comparatively unresponsive to this class of drug. Although opioids offer an alternative and are probably a more potent option than NSAIDs, they tend to be avoided in behavioural studies due to their non-specific (confounding) effects upon normal behaviour. Drugs such as buprenorphine are known to increase activity levels [19]–[21], however, more subtle behaviours that could be caused by pain might be less affected.

As we have done previously, this study utilised a multi-faceted approach using both manual and automated behaviour analyses, and measured the endocrine stress response to surgery (by non-invasive faecal corticosterone assay). We investigated the effects of buprenorphine in 2 mouse strains (C57BL/6Crl and C3H/HeNCrl) in an attempt to distinguish between its analgesic and non-specific effects. We wished to establish if pain-specific behavioural scoring under these circumstances provided a practically useful means of evaluating the post-surgical welfare of mice. The results showed buprenorphine can effectively relieve pain in mice at a dose rate similar to that currently used in other species, but this relies on applying an appropriate method of behaviour scoring. Pain-specific scoring was more successful than activity monitoring due to fewer confounding effects on behaviours thought to be specifically linked to pain.

## Materials and Methods

### Ethics Statement

Mice underwent vasectomy as part of an on-going transgenic mouse breeding program. No mice were used solely for the purpose of assessing pain. All procedures were approved by the UK Home Office (Project Licence PPL 60/3793) and by the Newcastle University Animal Ethics Committee. The work also complied with the guidelines of the Committee for Research and Ethical Issues of the International Association for the Study of Pain (IASP).

### Animal Husbandry and Study Design

Inbred male mice (C57BL/6JCrl, n = 24 and C3H/HeNCrl, n = 24) aged 10 weeks on arrival were obtained from a commercial supplier (Charles River, Margate, UK). They were housed singly upon arrival (Type MB2 cages, Techniplast UK Ltd, Northants., UK) for 2 weeks to acclimatise prior to the study. Sawdust was provided as bedding (Gold Chip, BS and S Ltd., Edinburgh, UK). Room temperature was maintained at 24±1°C with 15–20 air changes per hour. A 12 hour light-dark light cycle was used, with lights going off at 19:00 h. Food pellets (R and M No. 1, SDS Ltd, Essex, UK) and water were provided *ad-libitum*. Animals were weighed daily for 1 week prior to surgery, on the day of surgery, and on the day following surgery. Mice were randomly allocated into six groups of eight (four of each strain per group). Three groups underwent vasectomy (‘V’) via abdominal approach surgery. Once anaesthetised, these were given a subcutaneous injection of either saline (group VSa), or one of two doses of buprenorphine (‘Bup’ 0.01 or 0.05 mg/kg; low dose group VBupL and high dose group VBupH, respectively). Three sham groups; one receiving saline, one low dose buprenorphine and one high dose buprenorphine (groups ASa, ABupL and ABupH, respectively) did not undergo surgery but underwent all of the same preparations as the mice that underwent surgery, including anaesthesia (indicated by ‘A’ in the treatment acronym). A randomised block design ensured treatment groups were randomised with respect to surgery day and the time of surgery.

### Experimental Procedure

Mice were moved to the operating theatre before surgery began, and injections were given subcutaneously in this room following induction of anaesthesia with 5% isoflurane in 5 L/min oxygen. The fur on the lower abdomen was shaved and the skin sprayed with chlorhexidine (Hydrex Derma Spray, Adams Healthcare, Leeds, UK). Surgery began at 08:00 hours, with the same treatment-blinded surgeon operating on all mice. Anaesthesia was maintained with 2–2.5% isoflurane in 1 L/min oxygen after mice were individually placed on bedding (‘VetBed’, Kennel Needs and Feeds, Morpeth, UK) on a heating blanket (Harvard Apparatus, Edenbridge, Kent, UK) to maintain body temperature. Surgery began with a 1.5 cm transverse incision through the skin and abdominal muscles. The vas deferens was identified for both testes and a portion (approximately 0.5 cm in length) of each vas deferens was removed using cautery. The muscle and skin were closed separately with 5/0 polyglactin 910 (‘Vicryl’, Ethicon Ltd, Edinburgh, UK). Tissue glue (‘Nexaband’, Abbot Laboratories, Queenborough, Kent, UK) was also applied to ensure closure of the skin incision. In sham treated mice the surgical site was shaved and chlorhexidine applied, and anaesthesia was maintained for a period equivalent to that needed to complete surgery in the other vasectomised groups (∼15 minutes). Following the completion of surgery or the sham procedure, animals were transferred to an incubator maintained at 28±3°C where they were closely monitored for approximately 1 hour. Following this recovery period they were transferred to a quiet adjacent room for filming of post-operative behaviour.

### Filming and Behaviour Data Collection

The data intended for manual analysis were collected by filming each mouse for 6 minutes in a clear polycarbonate cage (Type 1144B, Tecniplast UK Ltd, Northants., UK) against a blue background, using a digital video camera (Sony DSR-PD1P, Japan) and ‘MiniDV’ tapes. A camera operator remained in the room and used a tripod to closely track movements so that subtle activities would be observable later. The mice were filmed again to obtain data for analysis with the automated analysis system HomeCageScan (HCS; Clever Systems Inc., USA). For this they were placed into an identical adjacent cage and filmed for a further 20 minutes, but with the camera fixed to the tripod and set at 30 cm from the cage front. The operator left the room whilst this footage was obtained. Filming cages contained sawdust bedding (approximately 3 cm deep) but no food or water. These were not necessary as experience showed such short behaviour assessments virtually exclusively involve exploratory behaviour irrespective of whether consumables are provided. A separate advantage of this was that it prevented the food hopper or water bottle obscuring the subject. The fixed camera position was essential to successful use of HCS in providing an unchanging background and preventing extraneous reflections (as occurs with a person present). Cages were wiped clean with 70% ethanol, allow to dry, and bedding was renewed before filming mice from different home-cages. At the end of filming mice were returned to their home-cages and kept in a warm room (24±1°C) for 24 hours to further aid recovery. We did not collect any baseline (pre-surgery) behaviour footage as the main focus was on evaluating treatment differences, not the effect of repeated exposure to the filming cages. Collecting and analysing these data would also have been unduly time consuming.

### Behaviour Data Analysis

The initial (6 minute) behaviour recordings were analysed by one treatment-blinded observer using ‘The Observer Video Pro’ software (Version 5.0, Noldus Information Technology, Wageningen, NL). The frequency, and where appropriate the duration, was calculated for each behaviour. Behaviours in the manual analysis were selected from a previously established ethogram [2] with the addition of one subsequently recognised and potentially relevant act; ‘lie flat’.

The remainder of the behaviour data (20 minute segments) were processed for automated analysis with HCS. Tapes were first digitised in MPEG1 format and then transferred to a computer running HCS software. The HCS system can be used either ‘online’ or ‘offline’ depending on the software version used. It computes the occurrence of up to 38 pre-programmed mouse behaviours. As with other versions, our ‘offline’ version (HPT3.0) allowed data to be analysed at twice normal rate, hence results were still obtained relatively rapidly.

### Faecal Corticosterone Assay

Faeces were collected from individual mice 9 hours after surgery. This was in line with previous observations that this constituted the time of peak corticosterone excretion following vasectomy in the strains of mice being investigated [2]. Baseline samples were taken on the day prior to surgery, exactly 24 hours before post-operative sampling. Baseline samples were collected in the holding room, and post-operative samples were collected in a room adjacent to the post-surgery recovery room. Faecal samples were stored at −80°C prior to processing. Corticosterone was extracted from the faeces using methanol. Briefly; 1 ml of 90% methanol was added to each sample. These were then homogenised using a mechanical homogeniser for 20–30 seconds and subsequently vortexed at 22°C and 1400 rpm for 30 minutes. The mixture was then centrifuged for a further 10 minutes at 2500×g. The supernatant was removed and dried in a Speedvac for 2–4 hours at 45°C. The dried samples were stored at −80°C overnight; re-suspended the following morning in 1 ml Dulbecco's Phosphate Buffered Saline, and vortexed for 1 minute at 22°C. Hormone levels were determined using the IDS OCTEIA Corticosterone HS Enzyme immunoassay kit (Immunodiagnostic Systems Ltd., UK) according to the manufacturer's guidelines.

### Statistical Analysis

All statistical analyses were conducted using SPSS (SPSS, Chicago, USA). The pre-operative body weights from the morning of surgery (baseline values) were compared between groups by univariate GLM with ‘Strain’, ‘Surgery’ and treatment ‘Group’ as fixed factors. Calculations of the mean percentage change in weight from baseline to 24 hours following surgery were then compared with the same between-group factors. *Post-hoc* tests (with *Bonferroni* probability correction) were used to highlight individual group differences.

The baseline and post-operative corticosterone data were analysed similarly. However, these data required log transformation to achieve normality. We determined the log change in corticosterone values from baseline to the 9 h peak value (log_10_(Peak/Baseline)) and compared this using the factors ‘Strain’ and ‘Surgery’, and then evaluated individual group differences as before (*Bonferroni*).

The behaviour data obtained manually underwent initial inspection using canonical discriminant analysis (DA). This was to determine the extent of behavioural differences and the individual activities contributing to any significant group separation. This technique effectively reduces large datasets by creating fewer new variables termed canonical discriminant ‘Functions’. The number of new discriminant Functions equals the number of groups minus 1. Individual behaviours are tested for the strength of their correlation with each newly created DA Function, so allowing the identification of those principally responsible for group differences. This, therefore, provided a convenient means of identifying activities as either individually important or more appropriate for computing composite (summary) scores. Analysis of the composite scores and other behavioural outcomes of DA was undertaken using a General Linear Model (GLM), again with ‘Surgery’, treatment ‘Group’ and ‘Strain’ as between-subjects factors. *Post-hoc* tests (*Bonferroni*) were again also used to identify individual differences according to treatment type and strain.

The data collected with HCS underwent a similar analysis methodology, although the individual activities used differed from those in the manual analysis. This was an unavoidable consequence of the two data collection methods. HCS is ‘hard-wired’ to the various elements of behaviour that the system scores, whereas in manual analysis the user can include whichever behavioural elements they so wish.

Finally, Pearson's correlation was used to determine if/how the various individual or composite behaviour scores determined from the manual and automated scoring methods correlated with peak corticosterone values. All results in the text and Table 1 are means ±1 Standard error (SEM). Unless stated otherwise all figures show mean value +1SEM. Corticosterone units are ng/g faeces. *Alpha* level for all analyses was 0.05.

**Table 1:** The results of manual behaviour analysis showing the mean (±SEM) frequency (counts) or duration (seconds) of behaviours selected using Discriminant Analysis to compute Composite behaviour scores.

Treatment	ASa		ABupL		ABupH		VSa		VBupL		VBupH	
Strain	C57	C3H	C57	C3H	C57	C3H	C57	C3H	C57	C3H	C57	C3H
Writhe	0±0.0	0.8±0.5	0±0.0	0±0.0	0±0.0	1±0.4	3.3±0.85	3.3±1.3	2.5±0.65	4.3±1.9	0.8±0.5	3.5±2.0
Press2	0±0.0	0.5±0.3	0.3±0.25	0.3±0.25	0±0.0	0.3±0.25	0.5±0.3	1±0.0	0.5±0.3	2.3±0.25	0±0.0	2.8±1.8
Rear Leg Lift	0±0.0	0.5±0.3	0.5±0.5	0.5±0.3	0.5±0.5	0±0.0	1.3±0.25	4.3±2.0	3±1.2	3.3±0.75	0.3±0.25	0.8±0.75
Lick Wound	2.8±1.2	0.3±0.25	0.5±0.5	1.3±0.5	1±0.7	0±0.0	17±2.3	7.8±1	19.5±4.1	4±1.3	2.5±0.25	0.5±0.3
*LW Duration*	*4.5±1.6*	*4.7±4.7*	*0.9±0.85*	*2.6±1.65*	*1.4±1.1*	*0±0.0*	*126.7±7*	*44.4±17*	*103.2±30*	*13.7±8.7*	*8.6±8.5*	*1.7±1.1*
Scratch Wound	0±0.0	0.5±0.5	0±0.0	0.3±0.25	0±0.0	0±0.0	0.3±0.25	6.5±2.7	0.3±0.25	7±2.3	0±0.0	2±2.0
*SW Duration*	*0±0.0*	*0.6±0.65*	*0±0.0*	*0.1±0.1*	*0±0.0*	*0±0.0*	*0.5±0.5*	*10±2*	*0.4±0.4*	*11.2±4*	*0±0.0*	*0.6±0.55*
Abnor Walk^(1)^	1±0.4	17±10.5	1.5±0.3	4.8±2.7	20.5±17	31.8±9.5	32±6	39.8±6.5	47.5±2.2	51.5±5.6	46.3±5	51.3±11.5
*AW Duration*	*2.6±1.35*	*24.1±12*	*3.4±0.95*	*9.8±5.5*	*67.5±55*	*73.9±22.5*	*91.6±18*	*97.6±19*	*143.4±19*	*139.4±27*	*280.9±11*	*160.7±45*
High Rear	21.5±7.5	31±3.8	23.5±2.6	23±1.2	3.5±1.8	1±0.7	3.3±1.8	8.8±2.1	0.8±0.25	0.8±0.5	0.5±0.5	2±1.2
*HR Duration*	*35.6±12*	*49.2±8*	*32.9±5*	*35.2±4.1*	*3.8±2.3*	*1.1±0.8*	*3.9±1.9*	*11±2.9*	*1.1±0.4*	*0.9±0.55*	*0.4±0.45*	*1.9±1.1*
Lick Head	12.5±0.5	9.3±2.5	8±1.2	5±0.7	8±2.4	7.8±1.6	6.3±1.6	1.8±0.5	6±2	1.3±0.25	3±0.7	2.5±0.3
*LH Duration*	*18.4±4.5*	*15.4±3.5*	*9.5±1.3*	*10.2±1.1*	*12.3±2.25*	*25.5±7*	*10.4±2*	*4.3±0.65*	*8.4±2*	*6.7±2.3*	*5.3±.85*	*8.8±1.1*
Scratch Head	1±0.4	2.3±0.85	1.3±0.5	0±0.0	0.5±0.5	0±0.0	0.8±0.5	0±0.0	0.3±.25	0±0.0	0.3±0.25	0±0.0
*SH Duration*	*0.6±0.25*	*1.1±0.4*	*0.9±0.3*	*0±0.0*	*0.4±0.35*	*0±0.0*	*0.6±0.45*	*0±0.0*	*0.4±0.45*	*0±0.0*	*0.5±0.45*	*0±0.0*
Scratch ‘Other’	0.8±.5	1±0.0	0.8±0.5	0.3±0.25	0.3±0.25	0±0.0	0.3±0.25	0.3±0.25	0.3±0.25	0±0.0	0±0.0	0±0.0
*SO Duration*	*0.5±0.3*	*0.8±0.15*	*0.7±0.4*	*0.3±0.25*	*0.2±0.16*	*0±0.0*	*0.3±0.35*	*0.3±0.35*	*0.3±0.35*	*0±0.0*	*0±0.0*	*0±0.0*
Dig	33.8±8	16.5±3.0	30.3±10	26.3±9.5	27.8±15.5	1.8±1.2	2.5±1.8	9.5±6.4	0.3±0.25	0.3±0.25	0.5±0.5	0±0.0
*DIG Duration*	*31.5±7.5*	*14.2±3.8*	*24.6±10*	*23.4±9*	*41.9±25.5*	*1.3±1*	*2.2±1.4*	*8.9±5.9*	*0.1±0.5*	*0.3±0.25*	*0.2±0.2*	*0±0.0*
Normal Posture	21.8±8.5	9.5±5.5	16±7	31.3±11	3.8±1.9	5.3±2.3	0.5±0.3	10.3±4.6	0.3±0.25	3.5±0.72	1±0.4	4.8±2.25
*NP Duration*	*195±72.5*	*43±25*	*196±75*	*171±52*	*249±62*	*187.6±6.5*	*7.7±4.9*	*77±34*	*4.9±4.9*	*234±80*	*82.3±65*	*251±61.5*
Walk Normal^(2)^	90±7	72.8±8.5	98.3±3.6	99.3±6.2	61.3±20.2	32.8±13.1	12.8±4.7	28.3±12	1.8±0.65	9.5±8.5	0.8±0.5	7±3.3
*WN Duration*	*155±13.5*	*105.3±8*	*199.3±13*	*145.8±7*	*162±57*	*97.9±47.6*	*24±9.3*	*50±19*	*5.2±2.7*	*20.5±16.2*	*6.2±0.55*	*13.4±8*
Stop^(2)^	92.5±7.5	98.5±3.3	94±6	99.8±2.8	73.3±7.1	73.3±8	65.5±4.3	73.5±59	70.5±2.6	67.3±0.65	49.8±9	60±13.5
*Stop Duration*	*139±16.5*	*190±7*	*118.4±14*	*167.9±8*	*72.1±2.8*	*154.6±25*	*98±13.9*	*143±20*	*92±15*	*167±22.5*	*58.4±10*	*170±45*

**Figure 1:**
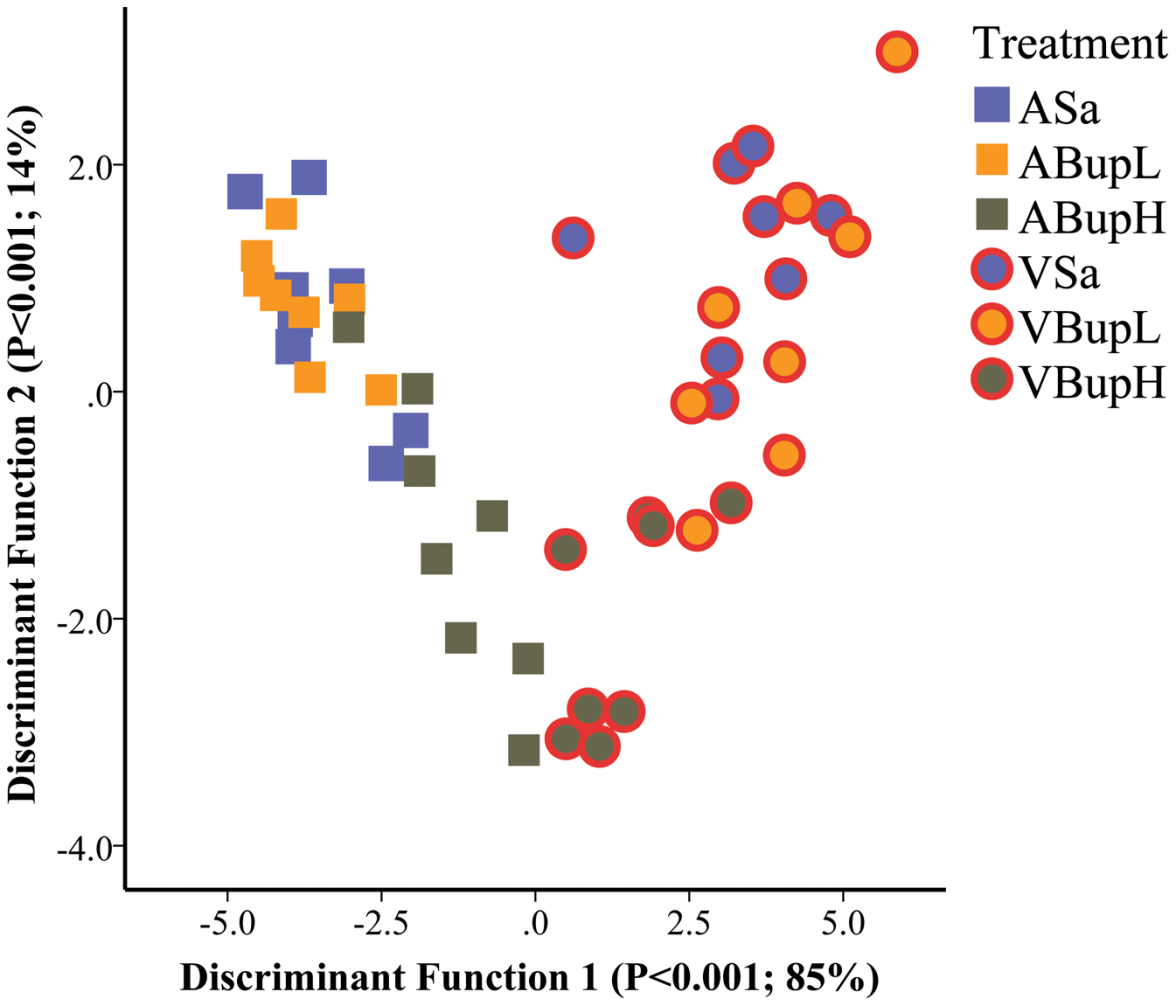
Plot of canonical discriminant scores for individual mice of each strain in each treatment group according to pain-specific behaviours scored 1 hour following anaesthesia or surgery (A or V; none versus red symbol borders). Mice were given saline (Sa; blue filled squares) or low or high dose buprenorphine (BupL (0.01 mg/kg) or BupH (0.05 mg/kg); indicated by orange or brown filled circles respectively). The significance of each Function in separating groups, and the % contribution to between-groups variance is given in brackets on each axis label. Note the significant separation depending on whether groups underwent surgery (Factor 1; p<0.001), and the effect of pre-surgery buprenorphine (VBupH; Function 2; p<0.001) resulting in the ‘V’ shaped convergence of individual scores; buprenorphine reduced abnormal behaviour to an extent similar to that seen in controls (groups ASa, ABupL, ABupH). (n = 8 per group).

The scores were used to distinguish between the behaviour of groups of C57 and C3H mice. Mice underwent treatment indicated as Anaesthesia ‘A’ or Vasectomy ‘V’ surgery and were given a pre-procedure s/c injection of saline (Sa) or High ‘H’ or Low ‘L’ dose Buprenorphine ‘Bup’. Four mice of each strain underwent each treatment type. Light shading indicates behaviours averaged to compute composite Score 1 (pain-specific measure); those shaded darker were used in Score 2 (Normal behaviour elements). Values in italics are duration of behaviour. Unshaded values were additional subset 1^(1)^ or 2^(2)^ components used in the DA (Figure 1) but not included in composite Scores 1 or 2.

## Results

### Surgery and Anaesthesia

The combined mean time from induction to completing surgery was 15±2 minutes and there were no unexpected complications or mortalities.

### Behaviour – Manual Analysis

Preliminary DA determined activities differing most in response to surgery. Activities were selected that positively or negatively correlated with the one significant Function produced by the analysis, with a cut-off R^2^ value of ≥0.1 or ≤−0.1 (Function 1; p<0.002, Wilk's Λ). Six behaviours that correlated positively with Function 1 (increased in response to surgery) and 10 that reduced (correlated negatively) were used to generate two subset (composite) scores (Subset 1 = +ve correlates; Subset 2 = −ve). These underwent additional analyses to evaluate the responses of the various groups to saline or drug treatment. Subset 1 was found to mainly comprise activities previously used as specific indices of post-surgical pain in rodents; wound licking, hind-leg stretching (‘rear leg lift’), an exaggerated form of pressing of the abdomen into the substrate while stretching (‘Press2’) and abdominal writhing. These were averaged to produce the composite measure ‘Score 1’. The final activity included in Subset 1 was abnormal walking, whereby mice showed either an unsteady or otherwise unusual stride pattern. This was not included in the averaged calculation of Score 1 as it occurred with excessive overall frequency compared to the other Score 1 behaviours. For a similar reason, 2 activities that showed the strongest negative correlation with Function 1 were also examined separately; Normal Walking (‘Walk Normal’) and periods of cessation of behaviour (‘Stop’) where mice were not moving but obviously not asleep. The other behaviours that correlated negatively with Function 1 were also those that would typically be classed as ‘normal’ and occurred at a frequency considered similarly acceptable (less than two-fold difference overall) to be used to create a second composite score; ‘Score 2’. This included high bipedal rearing, head and face washing (‘Lick Head’), digging, normal posturing and head/flank scratching. Activities were assessed for both their frequency and duration when appropriate (e.g. walking, grooming and rearing). However, with the exception of data on time spent inactive (Stop), the analysis found the duration data were no more effective than the frequency results for illustrating group and strain differences. As frequency measures are also more practicable for routine behaviour scoring the figures only show frequency results. However, the mean (±1SE) frequencies and, where appropriate the duration of all activities included in subsets 1 and 2, and the individual behaviour used to calculate composite Scores 1 and 2 are given in Table 1.

Scores 1 and 2 and the remaining subset 1 and 2 activities were then re-entered into a second discriminant analysis to determine how they separated the groups according to whether mice underwent surgery or only anaesthesia, or received a pre-procedure injection of saline or buprenorphine. With 6 groups, 5 canonical Functions were produced. Figure 1 is a scatterplot of the individual discriminant scores assigned to individuals on Functions 1 and 2 for mice in each treatment. Both Functions were found to be significant in terms of group separation (p<0.001, Wilk's Λ). As Figure 1 indicates, Functions 1 and 2 respectively explained 85 and 14% of the total variance. The group separation shows the major factor driving behavioural change was surgery, where Score 2 and other normal behaviours correlated negatively with Function 1 (Score 2, r = −0.59; Walk Normal, r = −0.46; Stop, r = −0.3), whereas Score 1 and Abnormal walking (pain-related behaviour) correlated positively (r = 0.6; 0.35, respectively) with this Function. Pain-specific behaviours (composite Score 1) correlated most strongly positively with Function 2 (r = 0.74). Overall, these various relationships between occurrence of normal and abnormal behaviour and how these were affected by surgery and buprenorphine treatment resulted in a ‘V’ shaped distribution of discriminant scores.

We hypothesised that Score 1 would increase in mice undergoing surgery relative to the non-surgery groups and would be lessened by pre-surgery buprenorphine, whereas the opposite would occur with Score 2; i.e. be greater in control mice and those given pre-surgery buprenorphine (if this effectively reduced pain).

**Figure 2:**
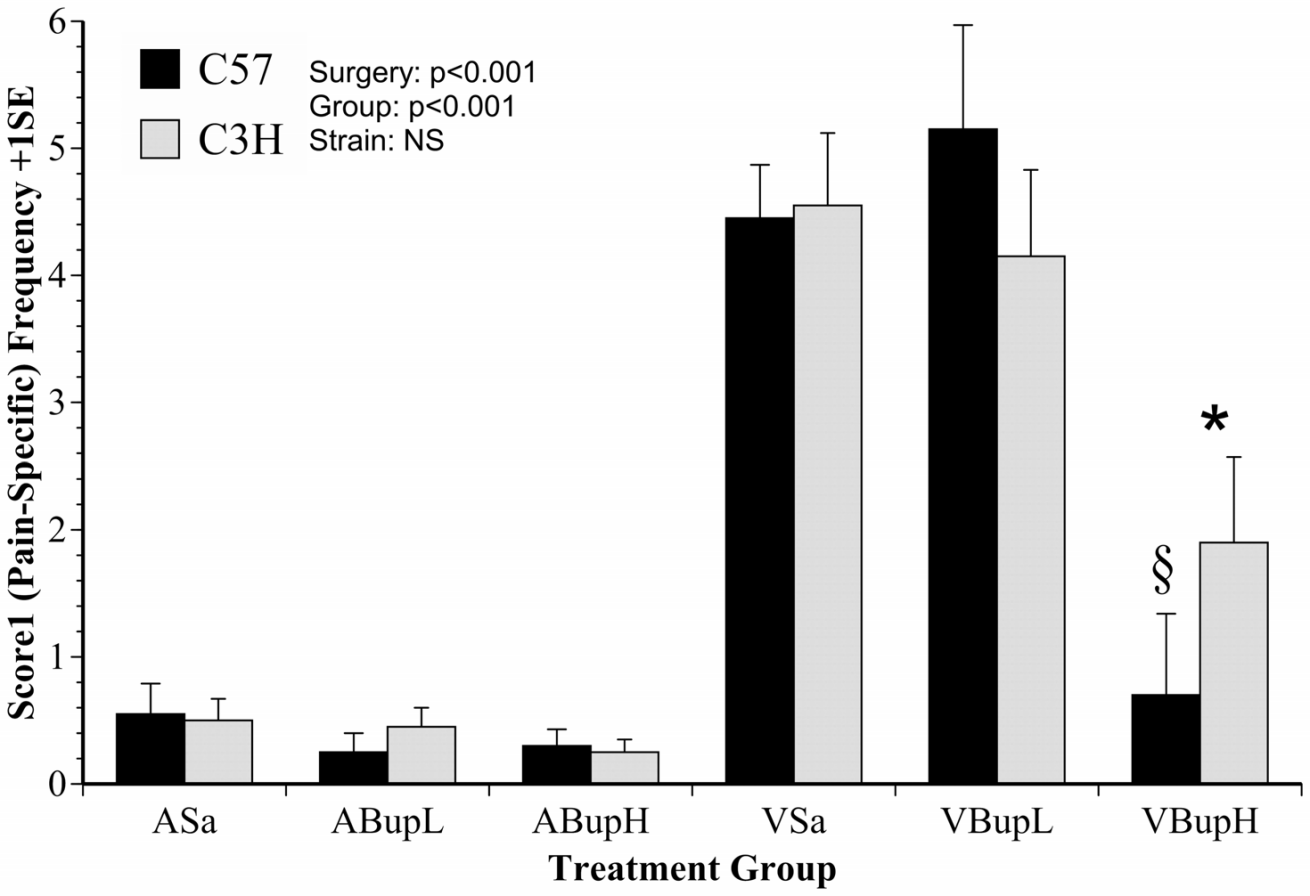
The mean frequency of the pain-specific composite behaviour measure ‘Score 1’ (+1SE) obtained via manual analysis. Behaviours indicating pain were not present in control groups given Saline (ASa), or low or high dose buprenorphine (ABupL, ABupH) but were significantly elevated in groups that underwent surgery following only Saline or 0.01 mg/kg buprenorphine (VSa, VBupL). The pain signs were attenuated by 0.05 mg/kg buprenorphine in both strains, but more so in C57 mice (n = 8 per treatment; comprising 4 of each strain). Symbols indicate results of *Bonferroni* comparisons: § C57 (VBupH) differs significantly from C57 mice in treatment groups VSa and VBupL (p = 0.008; p = 0.003, respectively); * Group VBupH differs significantly from VSa and VBupL (p<0.001).

As shown in Figure 2, Score 1 (pain-specific behaviour) was found to differ significantly between treatment groups (F(4,36) = 16.4, p<0.001), and surgery was the single most significant factor resulting in increased evidence of pain (F(1,46) = 56.3, p<0.001), but there were no overall strain differences in response to surgery (p = 0.8). *Post-hoc* analyses using these data found pain-related behaviour was highly significantly (p<0.001) more frequent in both the untreated vasectomised mice (VSa) and those given low dose buprenorphine (VBupL) than any other group, but these groups did not differ significantly from each other (hence no effect of low dose buprenorphine). The high dose surgery group (VBupH) showed significantly less Score 1 behaviour (indicating less pain) than both other surgery groups (P<0.001), to the extent that this pain-specific score 1 was equivalent (not significantly different from) each of the control groups. There were no significant differences between any of the control groups. However, high dose pre-surgery buprenorphine had a greater impact on C57 mice. These showed a greater reduction in pain-related behaviour than the C3H mice, with significantly reduced Score 1 frequency compared to either other surgery group (VSa, p = 0.008; VBupL, p = 0.003). The response of C3H mice was less pronounced, as comparisons with the relevant other C3H surgery groups were not significantly different (but only marginally so in the comparison with VSa; p = 0.051).

**Figure 3:**
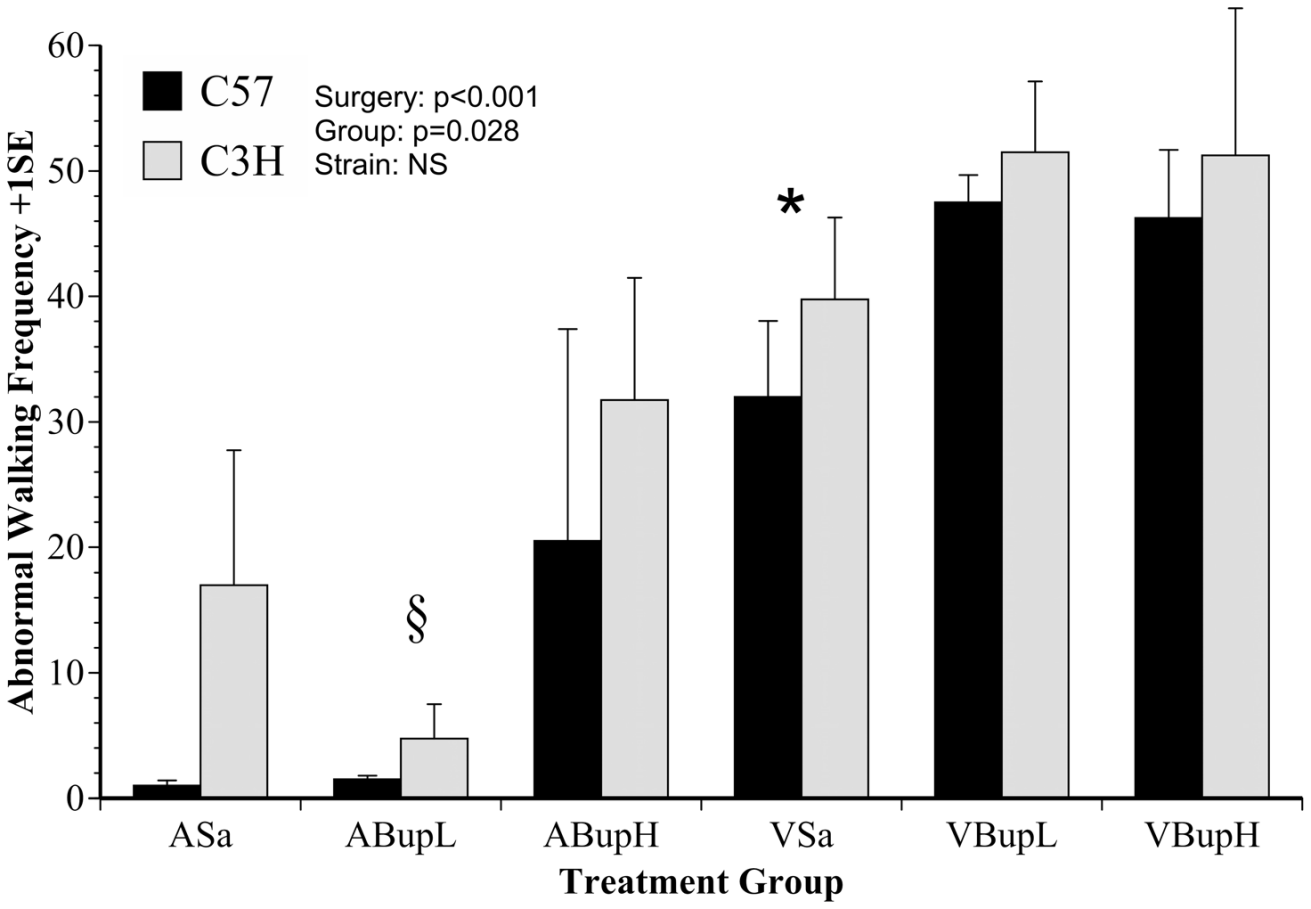
The mean frequency of abnormal walking behaviour (+1SE) in C57 and C3H mice in each control and surgery group. High dose buprenorphine significantly increased abnormal behaviour frequency in controls (group ABupH) making it impossible to determine any post-surgical analgesic effects. (n = 8 per group; 4 of each strain). Symbols indicate individual group comparisons: * Group VSa cf. Asa, ABupL (p = 0.031, 0.004, respectively); § Group ABupL cf. VSa, VBupL, VBupH (p = 0.004, <0.001, <0.001, respectively).

Figure 3 shows the frequency of abnormal walking (identified as pain-associated by DA). The analysis showed an overall ‘Group’ effect (F(1,36) = 3, p = 0.028), indicating the various treatments significantly affected this activity. Abnormal walking appeared to be somewhat more frequent in C3H mice, but ‘Strain’ was not significant. As Figure 3 illustrates, the surgery factor was also significant (F(1,46) = 412, p<0.001) as abnormal walking was increased in all surgery groups. *Bonferroni* analysis showed significantly more frequent abnormal behaviour in the untreated surgery group (VSa) than in either the untreated control group (ASa; p = 0.031) or in controls given low dose buprenorphine (ABupL; p = 0.004), but not compared to the high dose controls (VBupH) where abnormal activity also increased (possibly indicating non-specific (confounding) effects of buprenorphine on this behaviour). This was not seen following low dose buprenorphine, where, like the other control groups, there was significantly less abnormal walking behaviour than in any of the surgery groups (p = 0.004, <0.001, <0.001; for comparisons of ABupL with groups VSa, VBupL and VBupH, respectively). There were also no significant differences found between any of the individual surgery groups and no individual difference in the responses of the two strains either to surgery or the control treatments. Although high dose buprenorphine seemed to increase abnormal walking in C3H controls, relatively large variation rendered this comparison non-significant also.

**Figure 4:**
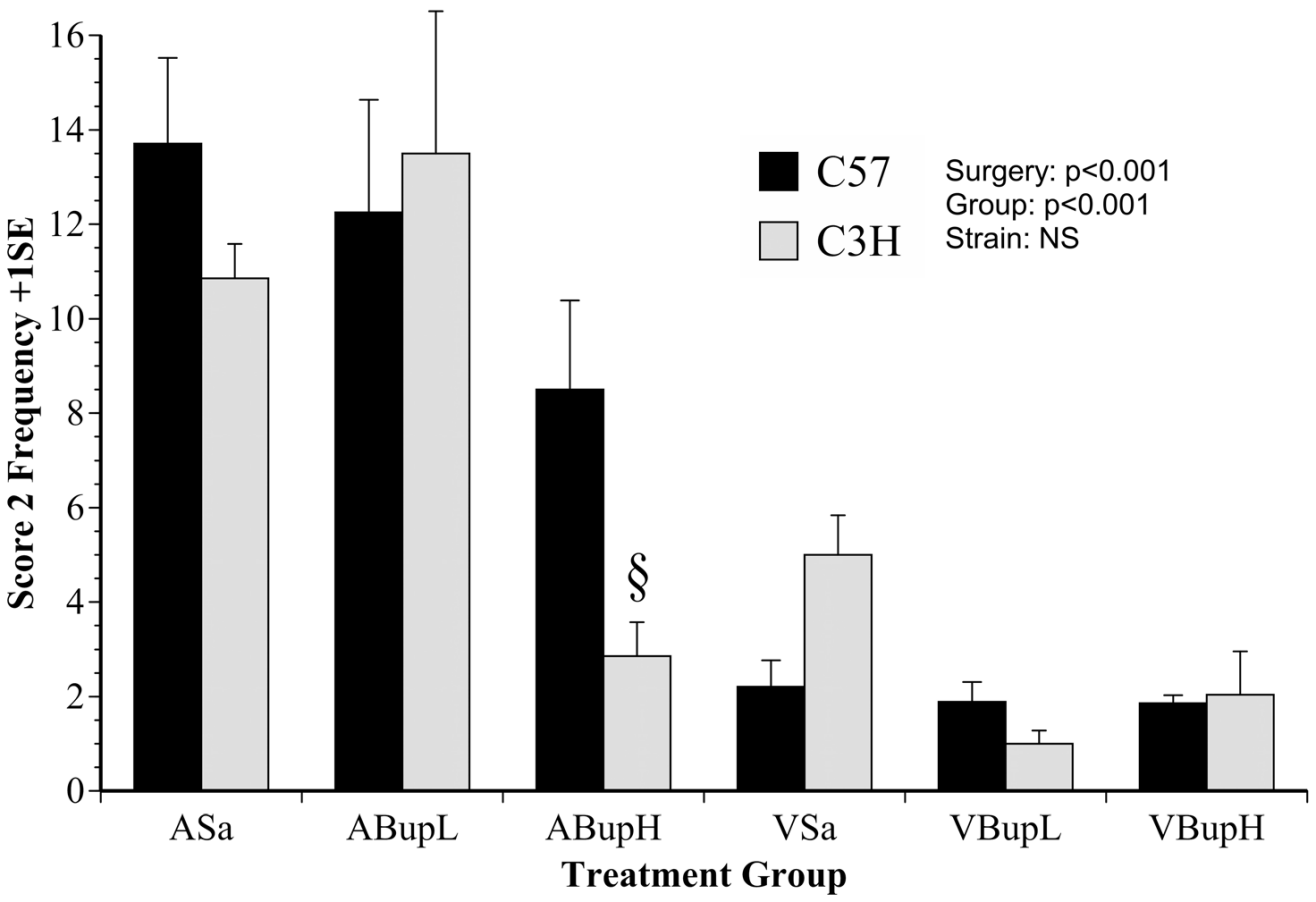
The mean frequency of the Composite normal behaviour measure ‘Score 2’ (+1SE) obtained from manual behaviour analysis. This was computed from the occurrences of bipedal high rearing, head and face washing (‘Lick Head’), digging, normal posturing, head scratching or of other body regions (e.g. flank). Note the significant reduction in normal behaviour caused by buprenorphine in both strains (ABupH), which was significant in C3H mice in this group compared to both other control groups. (n = 8 per group; 4 of each strain). Symbols indicate individual group and strain results whereby: * Group ABupL, Score 2 significantly greater frequency than all other groups except Asa (p≤0.001); § C3H mice in group ABupH vs. C3H groups Asa, ABupL (p = 0.039, 0.008, respectively).

Three summary measures of normal behaviour were tested; composite Score 2, Walk Normal and Stop. The frequency of Score 2 is shown in Figure 4. The relative occurrence of Score 2 in the various groups was virtually the reciprocal of the abnormal walking frequency shown in Figure 3, but here (Figure 4) occurring significantly more overall in the non-surgery versus surgery groups (F(1,46) = 52, p<0.001). There was also a significant overall treatment effect (F(4,36) = 8.4, p<0.001), but again no overall strain difference. Unsurprisingly, behaviour was relatively normal in the untreated controls (ASa), and as seen with Score 1 and abnormal walking, the low dose of buprenorphine again had little impact on controls (group ABupL). The mice in the latter group also showed more normal behaviour than any other group (p≤0.001 for all *post-hoc* comparisons) except group ASa. However, high dose buprenorphine significantly impacted by reducing the normal behaviour of controls (ABupH) to the extent that this group was not significantly different from any of the surgery groups (p>0.08 for each *Bonferroni* adjusted comparison). This effect of buprenorphine was especially apparent in C3H mice. Those in the low dose control group (ABupH) showed significantly less normal behaviour than both other C3H control groups (ASa, p = 0.039; ABupL, p = 0.008). Notably, the high drug dose given to control C3H mice caused as great a reduction in normal behaviour as that caused by surgery alone, or either surgery group given buprenorphine. By contrast, the depressing effect on normal behaviour in C57 mice was relatively minor, and the behaviour of the C57 mice in group ABupH was not significantly different from that in the other C57 control groups (ABupH C57 compared to ASa or ABupL; p≥0.15 in each case). Compared to Score 2, normal walking occurred with a considerably greater magnitude overall, but strain effects and the pattern of response according to treatment were so similar to Score 2 that it was unnecessary to depict the results graphically. They can be found in Table 1.

**Figure 5:**
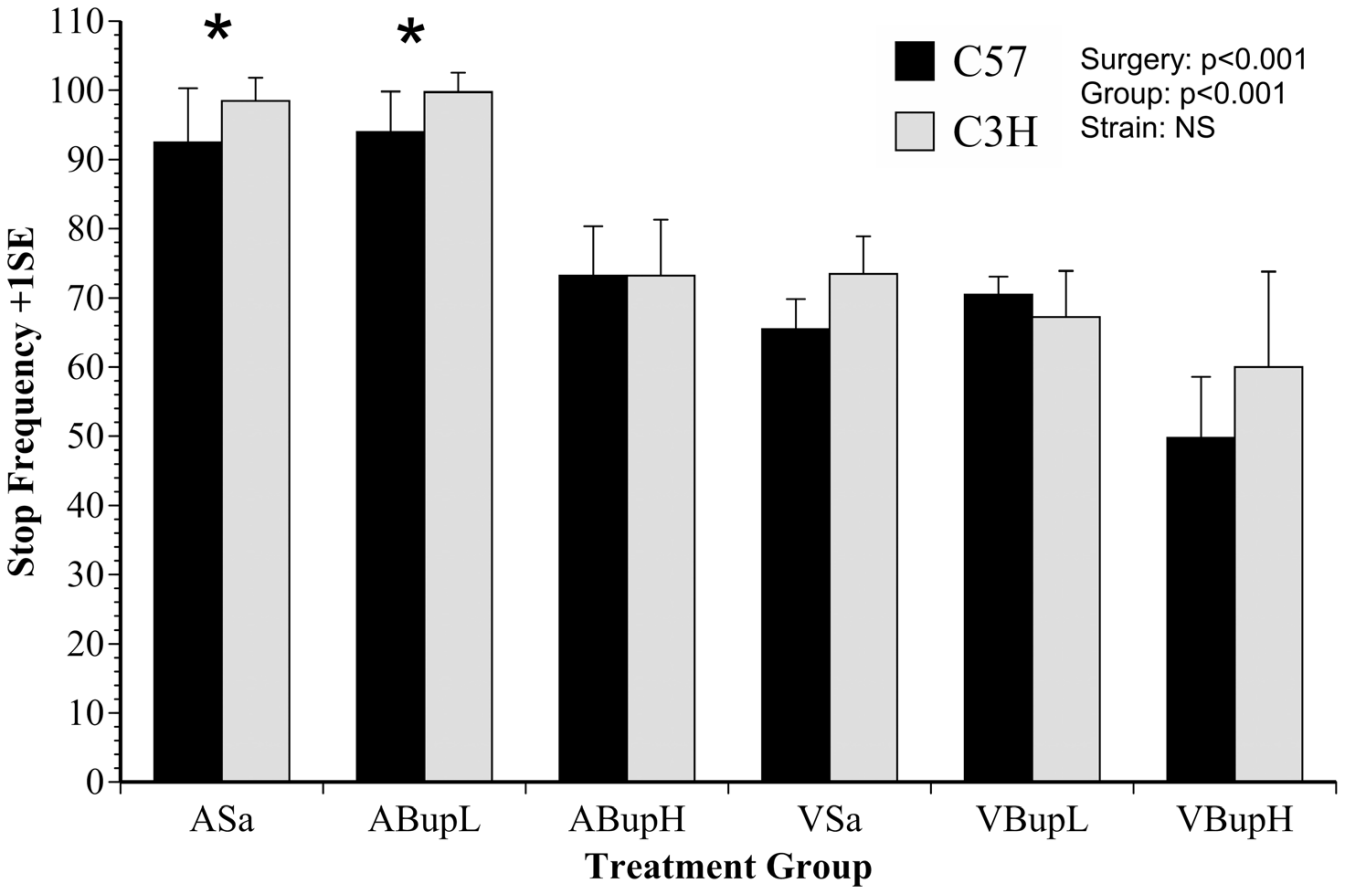
The mean frequency of inactive periods ‘Stop’ (+1SE) in each control and surgery group in data collected manually. Buprenorphine resulted in a decrease in periods of inactivity both in the control and surgery groups given the high dose of drug. A similar decrease in inactivity also occurred in the other two surgery groups (VSa and VLBup). (n = 8 per group; 4 of each strain). Asterisks indicate groups Asa, ABupL were significantly more frequently ‘Stopped’ than all other groups (p≤0.031 for all individual comparisons).

Surgery significantly reduced the overall frequency of inactivity (‘Stop’) (F(1,46) = 28, p<0.001; Figure 5) so mice were overall more active. There were no significant overall strain differences, but ‘treatment’ was significant (F(5,42) = 12, p<0.001). *Bonferroni* comparisons showed this arose because high dose buprenorphine reduced the frequency of inactive periods in both the control and surgery groups (ABupH and VBupH). These groups did not significantly differ from each other, and the high dose control mice were significantly more active (‘Stop’ further reduced) by comparison with both of the remaining control groups (ABupH vs. ASa, ABupL; p = 0.031, p = 0.017, respectively) who were more frequently inactive than all other groups (p≤0.031 for each individual comparisons). There were no significant effects of surgery or overall effect of treatment on time spent inactive (Stop *duration* as opposed to frequency; Table 1). However, in line with the frequency results, untreated control mice (ASa) spent more time inactive than most other groups, including the low dose drug controls (ABupH, p = 0.017), the untreated vasectomised mice (VSa, p = 0.037) and the high dose surgery group (VBupH, p = 0.019). These effects, however, were mainly due to the greater magnitude of changes in C57 mice. Overall these mice spent considerably less time inactive (‘Stopped’) than the C3H mice (‘Strain’ factor significant; F(1,36) = 35, p<0.001), and the main contributors to this overall difference were the C57 groups ABupH, VSa, VBupL and VBupH who spent a comparatively shorter time inactive.

### Behaviour – Automated Analysis (HCS)

**Figure 6:**
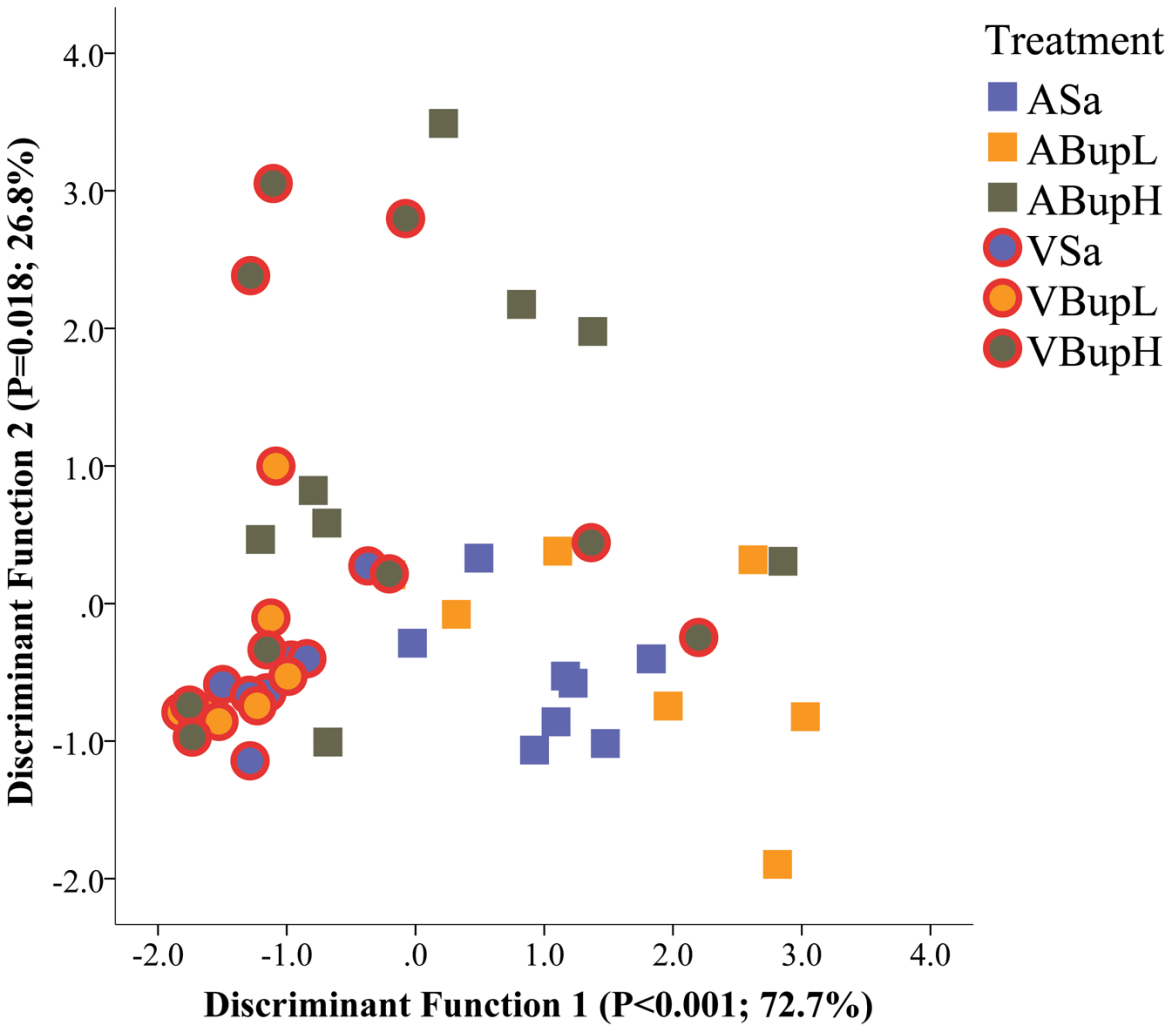
Plot of canonical discriminant scores for individual mice in each group according to Low, Medium and High frequency normal behaviour data from analysis with HCS (Automated). Groups are identified as in the Figure 1 legend; the axis labels show the significance of each Function and its respective percentage contribution to between-group variation. Although activity changes showed significant separation of the surgery and control groups (Function 1; P<0.001), compared with the results of pain scoring there was a lack of differentiation according to whether mice were treated with saline or buprenorphine. Low frequency measures were: ‘Jumping’ and ‘Come Down’; Medium frequency: ‘Rear Up’, ‘Walk Left’ and ‘Walk Right’; High frequency: ‘ComeDown from and to Partially Reared’ (2 behaviours), ‘RearUp from and to Partially Reared, ‘Remain Low’, Remain Partially Reared’, ‘Sniff’ and ‘Walk Slow’. (n = 8 per group).

The analysis registered 29 of the 38 behaviours the HCS system can record. As in the manual analysis, these were entered into DA with surgery as the between-subject's grouping variable. This produced one significant canonical Function (Function 1; p = 0.002, Wilk's Λ). Twelve variables (behaviours) that met the R^2^ cut-off previously applied (≥0.1/≤−0.1) all correlated positively with this Function (i.e. reduced in response to surgery). Grooming and stationery (Stop) periods were the only variables that correlated negatively with Function 1 (increased following surgery) but only grooming met the R^2^ cut-off so ‘Stop’ was excluded. The positively correlated variables were grouped according to whether they occurred with Low, Medium or High frequency. This was because their respective averaged frequencies (over all groups) closely approximated to 10, 50 or 100 within each 20 minute recording. The low frequency group included: ‘Jumping’ and ‘Come Down’; medium frequency activities were ‘Rear Up’, ‘Walk Left’ and ‘Walk Right’, and the high frequency group included ‘ComeDown’ from and to ‘Partially Reared’ (2 behaviours), ‘Rear Up’ from and to ‘Partially Reared’, ‘Remain Low’, ‘Remain Partially Reared’, ‘Sniff’ and ‘Walk Slow’. The Low, Medium and High summary measures were then calculated. These composite scores and grooming were used in an additional DA analysis to determine how group behaviour characteristics differed according to treatment type. This produced two significant Functions (Function 1, P<0.001; Function 2, p = 0.018, Wilk's Λ) that together explained 99.5% of the between-groups variance (72.7 and 26.8%, respectively). Figure 6 shows a scatter plot of the Function 1 and 2 discriminant scores assigned to individual mice in each treatment group. The Low and High frequency activities were most strongly positively correlated with Function 1 (R^2^ = 0.96, 0.76, respectively), whereas the Medium frequency activities correlated positively with Function 2 (R^2^ = 0.7) and grooming was negatively correlated with Function 1 (R^2^ = −0.52). As Figure 6 illustrates, there was a comparatively greater dispersion of discriminant scores relative to those obtained in the manual analysis (Figure 1) with several mice in the surgery groups overlapping with controls. The principal outliers were those given high dose buprenorphine; 2 of which were assigned a disproportionately higher score on Function 1, and 3 mice on Function 2 compared to the other mice undergoing surgery. The results were further summarised by calculating a geometric average of the results across the 3 behaviour categories (G^behave^ = 10∧((Log10(Low+1×Medium+1×High+1)/3)−1) to provide a general activity score. This was then compared between groups using ANOVA with the factors previously described.

**Figure 7:**
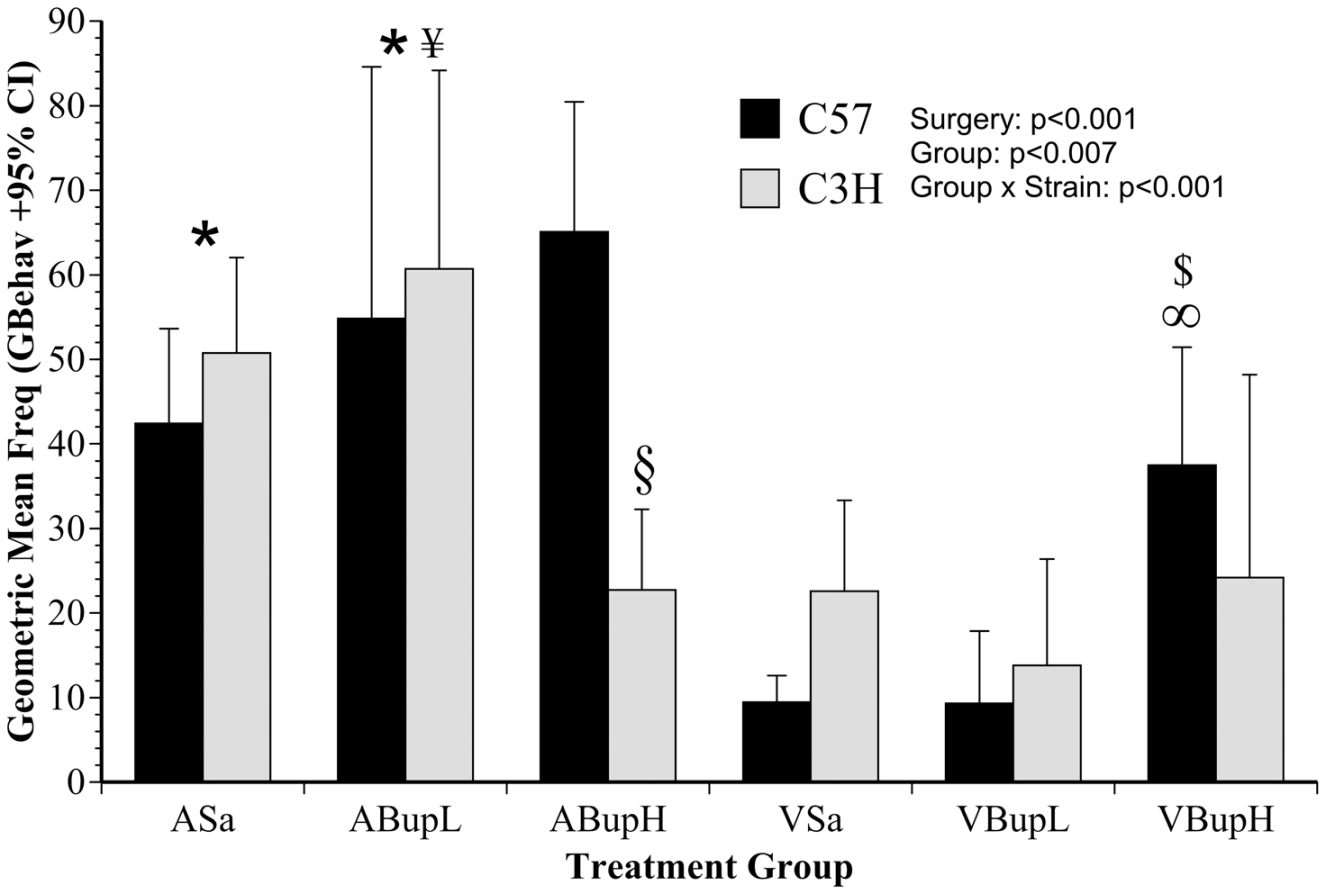
Geometric mean frequency of the composite normal activity measure (G^behave^) from HCS (automated) analysis (with 95% confidence intervals). The text gives details of how this was computed from the Low, Medium and High activity scores. Results were similar to those obtained manually; surgery reduced normal behaviour incidence and C3H mice were more susceptible to the confounding effects of high dose buprenorphine; i.e. normal behaviour was significantly depressed in this strain (group ABupH). (n = 8 per group; 4 of each strain). Symbols indicate results of individual group comparisons: * Groups Asa, ABupL vs. VSa, VBupL (p<0.001 for respective comparisons); ¥ Group ABupL vs. VBupH, p = 0.002); § C3H mice in group ABupH cf. Asa, ABupL (p = 0.01, p = 0.001, respectively); ∞ C57 mice in group VBupH vs. C57 mice in groups VSa, VBupL (p = 0.012, 0.011, respectively); $ C57 mice in group VBupH cf. C57 mice in group ABupL (p = 0.014).

Based on the distribution of discriminant scores (Figure 6) our *Ad-hoc* prediction was that G^behave^ would be reduced in surgery relative to non-surgery groups, with less clear strain and individual treatment effects. The mean frequency of G^behave^ (with 95% Confidence Interval) is shown in Figure 7. Surgery was the most influential factor unpinning behavioural alterations, and G^behave^ was significantly reduced in all vasectomised mice compared to controls (F(1,37) = 70.3, p<0.001). There were no overall strain differences, but treatment (F(4,37) = 4.2, p = 0.007) and the interaction term ‘Treatment x Strain’ were also significant (F(4,37) = 6.5, p<0.001). This was due to different responses to treatments, and the differential responses of C57 and C3H mice. The C3H mice tended to be more active following either saline or low dose buprenorphine, regardless of whether they underwent surgery. Primarily, however, the significant interaction arose because G^behave^ was only reduced by high dose buprenorphine in the C3H mice, whereas C57 mice in this group were relatively unaffected. Overall, the untreated and low dose controls (ASa, ABupL) were significantly more active than both the untreated and low dose buprenorphine vasectomy groups (p<0.001 for respective comparison with groups VSa and VBupL). The low dose buprenorphine controls were also more active than the high dose vasectomy group (ABupL vs. VBupH; p = 0.002). There were no overall group differences within the individual surgery or control groups, however, separate strain analyses showed high dose buprenorphine significantly reduced activity in C3H controls (ABupH) relative to the other C3H control groups given saline or low dose buprenorphine (p = 0.01, p = 0.001; cf. ASa, ABupL, respectively). This buprenorphine-driven reduction in activity was similar to that seen in all C3H mice following surgery. High dose buprenorphine had the opposite effect in vasectomised C57 mice where activity was significantly increased relative to either of the other pre-surgery treatments (VBupH vs. VSa and VBupL, p = 0.012, p = 0.011, respectively), but was significantly less in C57 mice compared to their respective control high dose group (VBupH vs. ABupH, p = 0.014).

**Figure 8:**
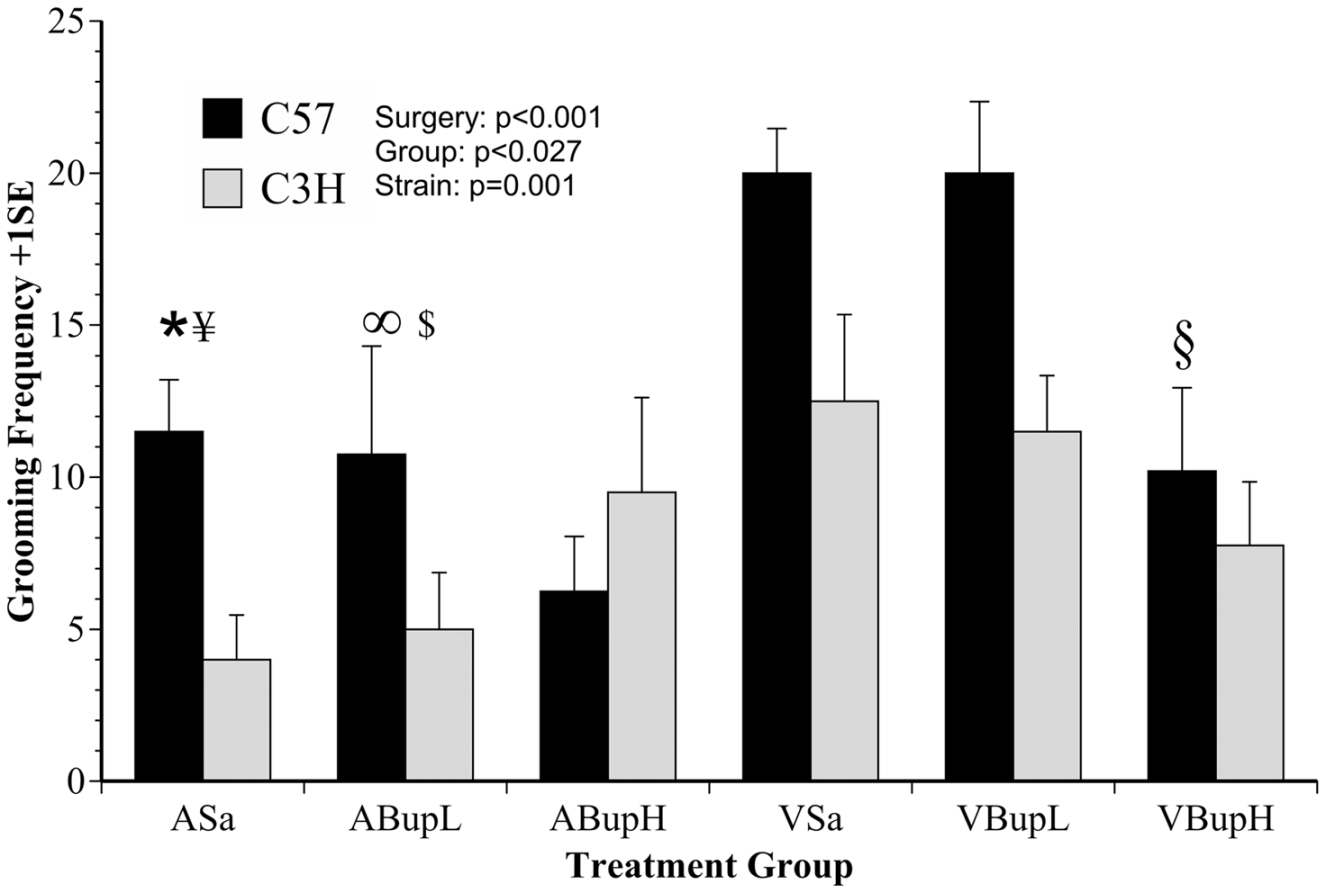
The mean frequency of grooming behaviour (+1SE) following automated behaviour analysis with HCS. Grooming increased in response to surgery but C3H mice groomed less overall. The C57 mice showed a greater increase following surgery which was significantly reduced by high dose buprenorphine. However, this effect was again confounded by a similar response in high dose C57 controls (n = 8 per group; 4 of each strain). Symbols show the results of individual comparisons: * Group ASa cf. VSa (p = 0.001); ∞ ABupL cf. VBupL (p = 0.002); ¥ ASa cf. VBupL (p = 0.002); $ ABupL cf. VSa (p = 0.001); § C57 mice in group VBupH cf. C57 mice in groups VBupL, VSa (p = 0.043, both comparisons).

The effects on grooming behaviour are shown in Figure 8, where HCS found changes in response to the various treatments were almost the opposite of those on general activity (G^behave^). Surgery caused a highly significant increase in grooming frequency (F(1,37) = 18.1, p<0.001), and the overall effect of treatment ‘Group’ was also significant (F(4,37) = 3.1, p = 0.027). Here, however, there was also a marked strain difference, as C57 mice groomed more overall (F(1,37) = 12, p = 0.001). There was a slight tendency for high dose buprenorphine to reduce grooming in C57 controls relative to the other saline or low dose control groups, but the reverse was apparent in C3H mice. Overall, buprenorphine had relatively little impact on grooming in either strain and no significant effects were found in strain comparisons between the various control groups. Surgery caused an increase in grooming and there was little evidence of a positive effect of buprenorphine when both strains were included in the analysis. However, the C57 mice not only groomed more overall, but the effect of high dose buprenorphine before surgery was also more obvious in these mice. Compared to the other surgery groups, the C57 mice in group VBupH showed a proportionately greater reduction in post-surgery grooming. There was little or no evidence of this in C3H mice. A one-way *post-hoc* ANOVA found no significant difference between the 3 pre-surgery treatments in C3H mice, but the C57 mice given high dose buprenorphine showed significantly less grooming compared to their counterpart groups given either low dose buprenorphine (p = 0.043) or saline (p = 0.043) before surgery. Additional *post-hoc* analyses (irrespective of strain) showed the saline and low dose buprenorphine control groups groomed less than their respective post-surgery counterparts: ASa vs. VSa (p = 0.001); ABupL vs. VBupL (p = 0.002), and all other comparisons between these groups were also significant (ASa vs. VBupL, p = 0.002; ABupL vs. VSa, p = 0.001). However, the two high dose buprenorphine groups did not significantly differ from each other (ABupH vs. VBupH, p = ns).

### Body Weight Changes

The body weight changes were similar between strains, both in terms of initial group variation and also in response to surgery. The data were therefore pooled. The pre-operative body weights of the mice did not differ significantly between the various treatment groups. The total average weight on the morning of surgery was 26.5±1 g. Surgery resulted in a significantly greater percentage weight loss over the 2 days relative to controls (F(1,46) = 17.8, p<0.001). The non-surgery mice showed a very slight gain (1.3±2 g) and post-surgery losses were relatively modest overall (−3.7±2 g). The treatment factor was also found to be significant due to the greater effect of surgery on weight change (‘Group’ factor significant; F(5,42) = 3.4, p = 0.01), however, the only individual (*post-hoc*) groups where weight loss significantly differed was in the anaesthesia control mice given low dose buprenorphine compared to the surgery group given the higher drug dose (ABupL vs. VBupH; p = 0.04). Overall, therefore, weight losses were not remarkable and so are not depicted graphically.

### Corticosterone Analysis

**Figure 9:**
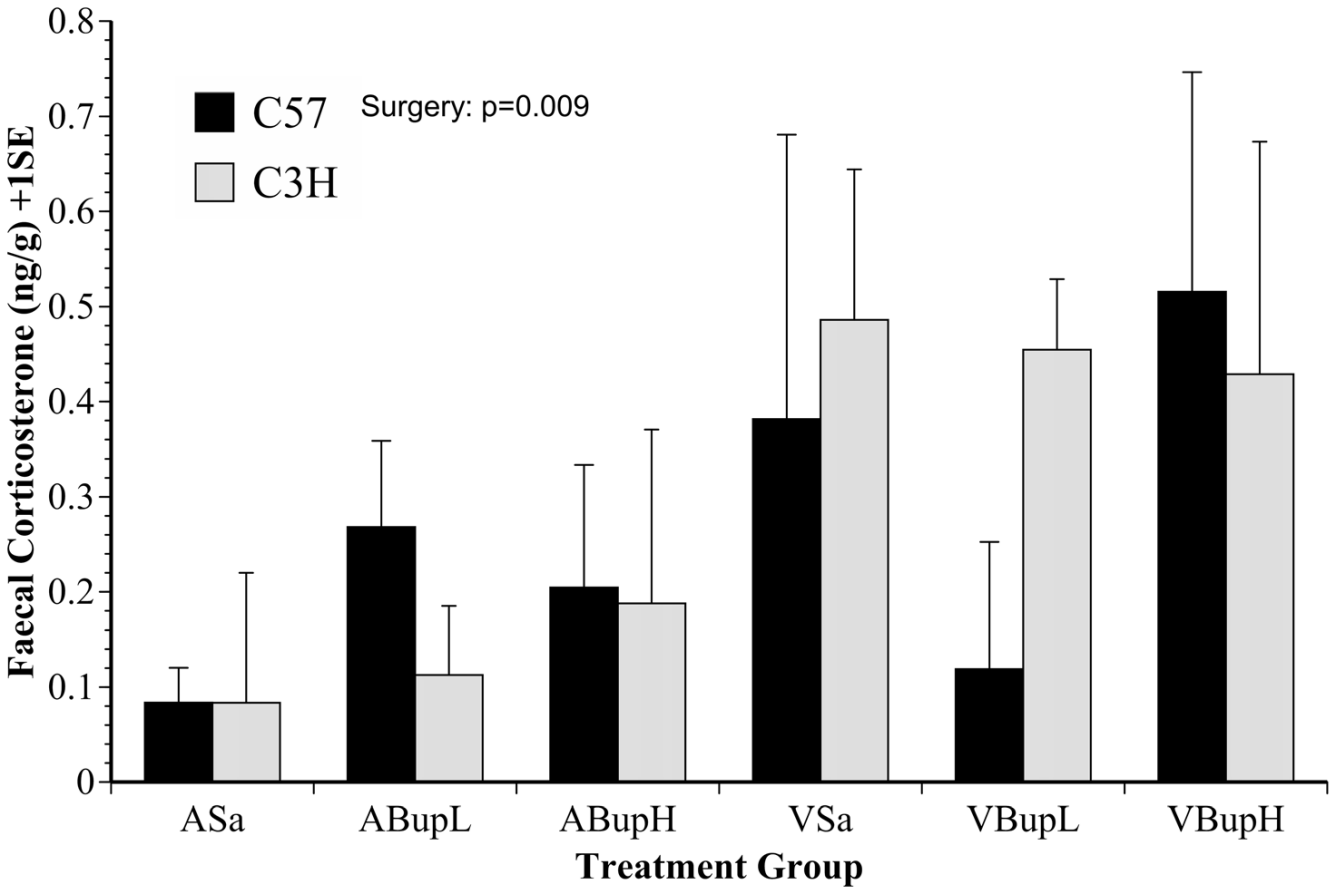
Corticosterone levels detected in the faeces of C57 and C3H mice 9(+1SE). Surgery caused an overall increase in corticosterone, but extensive variation rendered efforts to establish analgesic (stress reducing) effects of buprenorphine non-significant. (n = 8 per group; comprising 4 mice of each strain).

Two mice were excluded due to an inability to obtain valid faecal samples. Data from one other mouse in group VSa were also excluded based on a baseline corticosterone value of >800 ng/g; up to tenfold more than any other value recorded. The results are shown in Figure 9. The mean baseline (untransformed) corticosterone values were not significantly different between strains; 30.3±3.6 ng/g in C57 mice compared to 31.5±2.5 ng/g in the C3H strain. Overall, the corticosterone data were highly varied, as were the individual groups' responses to treatment. Corticosterone levels (transformed values; Figure 9) increased in all mice in response to all treatments, but more so in those that underwent surgery (F(1,43) = 7.4, p = 0.009). Although surgery in C57 mice also elevated corticosterone, the changes were especially variable in this strain and the overall significant ‘Surgery’ effect was primarily due to the increase in C3H mice (F(1,21) = 7.7, p = 0.011). *Bonferroni* analysis was again used to determine any individual group differences. The C57 mice showed a non-significant tendency to respond positively to low dose buprenorphine (VBupL), but levels were elevated both in this strain and also C3H mice following high dose buprenorphine (VBupH). Due to the highly varied nature of the corticosterone results there were no other significant findings to report. We assessed relationships between the various behavioural parameters and the results of corticosterone sampling. However, we could not find any significant relationships between the apparently enhanced stress response caused by surgery and any of the manually or automatically obtained behaviour results.

## Discussion

We investigated the effects of surgery and two doses of buprenorphine administered to C3H and C57 mice undergoing vasectomy. We hoped to detect signs of pain and to demonstrate positive (or otherwise) effects of buprenorphine on welfare. The study utilised both manual and computerised behavioural analyses and assessed faecal corticosterone levels. These methods were combined in a concerted effort to establish a more refined and practically useful approach to post-surgical pain assessment in mice than has so far been achieved.

We found pain-specific behaviours occurred following vasectomy, and scoring these was a more practicable and effective means of assessing pain and the analgesic effects of buprenorphine than general activity monitoring. Using this method we found buprenorphine given at 0.05 mg/kg (s/c) reduced pain, but was more effective in C57 mice. The C3H strain showed more pain-related behaviour overall, and were also more susceptible to the confounding effects of buprenorphine on behaviour. We found this to be the case irrespective of whether data were collected manually or using the automated system.

The ethogram used for the manual behaviour analysis was developed in an earlier study where we assessed the effects of meloxicam [2]. In that study, as here, we could not find any particular behaviour(s) whose occurrence effectively distinguished between treatments. As a result, we created summary (composite) scores to illustrate the main findings. The composite scores presently used were derived from the results of discriminant analysis (Figure 1) where we determined which behaviours were distinct between the surgery and non-surgery groups, and which differentially changed in response to the 2 test doses of buprenorphine. The inclusion of individual behaviours in each composite score was based on 2 qualifying criteria. Firstly, they required statistical relevance (in separating the control and surgery mice), but also potential for practical application such that they should be recognisable by even inexperienced staff. Score 1 included some activities previously identified as likely to be pain-related in both mice and rats [2], [15], and together with abnormal walking (which was assessed separately) provided our main pain-specific index. Score 2 was mainly comprised of normal activities (see Table 1), and grooming was also included as a normal activity.

The figures provide greater clarity concerning the outcomes of discriminant analysis and how the data were subsequently interpreted. As shown in Figures 2 and 3, the pain-specific index ‘Score 1’ and abnormal walking respectively increased following surgery, but unlike in our previous experiments, use of ‘pain-specific’ scoring also allowed us to identify significant dose effects. Whereas the lower dose of buprenorphine was not effective, the high dose (0.05 mg/kg) significantly attenuated the relevant behaviours (Figure 2). Furthermore, we were able to identify strain differences. Pain-specific behaviours were more frequent in C3H mice following high dose buprenorphine. Assuming these indicated pain, the C3H strain were therefore more painful than C57 mice. This conclusion is supported by our most recent findings wherein C3H mice showed a more elevated morphine conditioned place preference than C57 mice during development of bladder cancer [unpublished results]. That study also included automated analyses of behaviour and found that behaviour changes were also more extensive in C3H compared to C57 mice at the same stage of cancer development. These differential effects of high versus low analgesic dose rates detected by cage-side scoring, and the apparent greater sensitivity of one mouse strain compared to another confirm and strengthen our preliminary findings of similar differences using generalised activity [1], [2]. However, direct observation of pain-related behaviours seems to provide a more reliable and practicable means of detecting these differences in pain susceptibility. As such this approach is more likely to be useful in evaluating other mouse strains, other drugs, or other types of surgical procedure. The differences in responses to analgesic treatment in the two strains of mice could be due to a number of different factors. Different inbred strains of mice have been shown to vary in their nociceptive thresholds [22]. In the latter study C3H mice showed over double the response level compared to C57 mice in an abdominal constriction test. The ED_50_ of opioids such as morphine also vary considerably in different inbred strains of mice [23]. It is therefore likely that both genetic influences on nociceptive processing and differences in analgesic pharmacokinetics may contribute to the strain variation demonstrated by our study and others.

The results of manual activity scoring are shown in Figures 4 and 5 (Score 2 and inactivity; ‘Stop’ frequency) and those with the automated approach Figures 6, 7 and 8 (DA results, the summary activity measure G^behave^ and grooming, respectively). The findings obtained via automated data collection were generally more difficult to interpret than those obtained by pain-specific scoring. Figure 6 exemplifies this, and shows that the level of discrimination achieved between the various treatments was poor following discriminant analysis of data collected with HCS. This was mainly because, as was anticipated, buprenorphine had a range of confounding effects on general activity. For example, the high dose so adversely affected walking behaviour in controls (increased Abnormal Walking) t it impossible to assess any analgesic effects. Similarly, although surgery reduced normal behaviour frequency in the manual assessment (Score 2: rearing, digging, grooming, etc.; Figure 4), high dose buprenorphine also produced this effect in controls. These confounding effects of buprenorphine are relatively well known [19]–[21] and were also more obvious in C3H mice. The HCS results also showed that both surgery and buprenorphine significantly reduced our summary measure of normal behaviour (G^behave^) in controls, and the C3H mice were again most affected (Figure 7; group ABupH). As before, this made it impossible to establish the extent to which drug treatment *per se* contributed to the post-operative findings in the drug treated groups. Finally, all surgery groups and the controls given high dose buprenorphine were *more* active overall (‘Stop’ frequency decreased; Figure 5). The net consequence of this was that, by comparison with pain-specific scoring, the activity measures were less useful as markers of welfare. Such non-specific effects on behaviour may have been less pronounced had we been using NSAIDs rather than opioids. However, as previously mentioned, it seems likely that comparatively high (potentially toxic) dose rates of NSAIDs may be necessary to achieve effective pain relief in mice.

The buprenorphine dose rates were meant to be in the lower range still likely to provide effective pain relief, but minimise potential confounds. When given at 0.2 mg/kg (s/c) buprenorphine not only causes loss of body weight [24], [25], but also other unwanted behavioural effects that prevent reliable pain scoring [24]. More frequent dosing or use of a higher dose rate (2.4 mg/kg i/p) may not only adversely affect food and water consumption, but can also cause hyperthermia and hyperactivity [19]. In the present study all mice that underwent surgery lost weight. Although we could not detect any beneficial effect of buprenorphine in terms of preventing this, we avoided the adverse effects associated with higher dose rates. The choice of dose rate was also informed by a reported efficacy of 50% (0.01 mg/kg) and 100% (0.05 mg/kg) in the mouse abdominal constriction assay, and a 50% effect in hot-plate testing [26]. The limitations of these types of nociceptive tests are well known, and it can be hazardous to extrapolate from their outcomes in estimating therapeutic dose rates. The current study was an attempt to evaluate 2 relatively commonly used doses. Our data suggest the lower dose (0.01 mg/kg), in these strains of male mice, and for this surgical procedure, provided inadequate analgesia. Although the higher dose (0.05 mg/kg) had positive effects, it was probably also not completely effective. This is supported by the results of a recent study on the effects of vasectomy using the mouse grimace scale [13]. This showed a significant improvement with 0.05 mg/kg, with a possibly greater effect of 0.1 mg/kg (although this analysis was not reported by the authors). Unfortunately the present study was completed prior to development of the mouse grimace scale, and the quality of video recording, together with the method of filming meant our data were not suitable for retrospective analysis using this approach.

There was a clear trend to towards higher faecal corticosterone concentrations in untreated mice that underwent vasectomy. However, due to considerable variation in all treatment groups we were unable to detect any beneficial effects of either dose rate of buprenorphine. Baseline corticosterone values were very similar to those from a previous study [2], with samples taken at the same time point in normal control animals. It was therefore unlikely that the observed variable increase in corticosterone was a consequence of the sampling method or analysis. As similar variation has previously also been reported following surgery in both rats and mice [2], [5], [6], it seems corticosterone may be more effective as a stress marker than as a measure of post-procedural analgesic efficacy.

In previous studies of the effects of vasectomy in mice we reported on how automating behaviour analyses can reduce the time needed to conduct assessments without sacrificing relevance [1], [8]. However this work did not involve scoring pain-specific indicators, largely because the HCS system cannot detect them. Although originally marketed as a user-trainable system, the level of flexibility that would be needed to train the system to score these behaviours has not yet been achieved. Nevertheless, HCS remains a valuable tool to rapidly phenotype mice, such as in testing drugs that are supposed to affect motivation or anxiety. Until it has undergone further development it seems HCS has limited value in studies specifically evaluating pain.

Prior to undertaking this study our only convincing evidence of an effective treatment for vasectomy pain was reduced faecal corticosterone in mice given 20 mg/kg meloxicam. A considerably lower dose rate of meloxicam (1 mg/kg s/c) is effective in rats using a similar pain-specific approach to scoring [16], and buprenorphine at the commonly administered subcutaneous dose rate of 0.05 mg/kg also substantially reduces pain-specific rat behaviour [17]. As present results indicate buprenorphine given at 0.05 mg/kg was at least partially effective in mice, the apparent disparity in analgesic needs between rats and mice may not be so great when opioids are used.

## Conclusions

These data support the use of behavioural scoring as a means of assessing post-surgical pain in mice, but in particular, they indicate that assessing pain-specific behaviours is a more effective approach than general activity monitoring. The latter was more susceptible to the confounding effects of opioid administration, in this case caused by buprenorphine. The considerable individual variation seen in response to both surgery and analgesic administration supports a recommendation that dose rates should be adjusted in relation to the potential severity of the surgical procedure, the mouse strain, and the individual animals' response. This is likely to be achieved by assessing pain and procedure-specific abnormal behaviour changes. We would also recommend assessing multimodal analgesic regimens for post-surgical pain relief in mice, for example by combining buprenorphine and an NSAID. This could avoid the confounding effects likely to be associated with use of higher doses of buprenorphine (i.e. >0.05 mg/kg) but still improve the degree of pain control.
